# An expression profile analysis of ES cell-derived definitive endodermal cells and *Pdx1*-expressing cells

**DOI:** 10.1186/1471-213X-11-13

**Published:** 2011-03-01

**Authors:** Soichiro Ogaki, Seiko Harada, Nobuaki Shiraki, Kazuhiko Kume, Shoen Kume

**Affiliations:** 1 Department of Stem Cell Biology, Institute of Molecular Embryology and Genetics, Kumamoto University, Honjo 2-2-1, Kumamoto 860-0811, Japan; 2 The Global COE, Kumamoto University, Honjo 2-2-1, Kumamoto 860-0811, Japan

## Abstract

**Background:**

We developed an efficient *in vitro *method to differentiate mouse ES cells into the definitive endoderm (DE) and then Pdx1-expressing pancreatic lineages using mesodermal-derived supporting cells, M15. Using this method, resulting ES cell-derived DE and Pdx1-expressing cells were isolated by cell sorting, and their gene expression profiles were investigated with DNA microarray. Genes that were specifically expressed in DE and/or in Pdx1-expressing cells were extracted and their expression patterns in normal embryonic development were studied.

**Results:**

Genes whose expression increased in DE and Pdx1 positive cells compared to the undifferentiated ES cells were chosen and *in situ *hybridizations were performed. Out of 54 genes examined, 27 were expressed in the DE of E8.5 mouse embryos and 15 genes were expressed in distinct domains in the pancreatic buds of E14.5 embryos. Among those genes expressed were *Foxq1, CpM, Foxp4, Pcdh1, and Zmiz1*, which were previously reported in other endodermal tissues. Genes, such as *Parm1, Tmem184a, Hipk2 *and *Sox4 *were reported to be expressed during early pancreatic development. *Nptx2, C2cd4b, Tcf7l2 and Kiss1r *were reported to be associated with beta cell or pancreatic functions in the adult. *Akr1c19, Aebp2, Pbxip1 *and *Creb3l1*, were novel and have not been described as being expressed either in DE or the pancreas.

**Conclusions:**

We identified 27 genes, including 4 novel genes expressed in DE and pancreatic progenitor cells during normal development using an ES cell *in vitro *differentiation system. These results showed that DE cells and Pdx1/GFP-expressing cells obtained from our M15 based differentiation method mimic cells during the normal developmental processes. Additionally, ES cells are an excellent model for studies of early developmental processes.

## Background

The endoderm gives rise to the respiratory and digestive organs such as pancreas, liver, lung, stomach, and intestine. These developmental processes are of great importance in therapeutics. The multipotent endoderm has the potential to be used to repair tissues. However, in spite of the importance of the definitive endoderm (DE) derived tissues, not much is known about how they emerge from the primary gut tube. Fate mapping studies suggest that the fate of the DE begins to segregate at embryonic day 6-6.5 (E6-E6.5) and that the progenitors that are fated to become specific tissues of the gut tube appear shortly after the completion of gastrulation [1,2]. The expression of the regional specific transcription factors has provided clues to how the endoderm is patterned into organ domains. In the chick, the progenitor for stomach, pancreas and intestine are segregated immediately after the completion of gastrulation. The progenitors receive inducing signals from the adjacent mesoderm during their migration and are specified before they reach their final destination [3]. *Pancreatic and duodenal homeobox gene 1 *(*Pdx1*) expression is by far the first sign of pancreatic differentiation detected at E8.5 in dorsal endoderm of the gut. *Pdx1 *is expressed before the buds become evident, and is required for the progression of pancreatic and rostral duodenal development [4]. Genetic lineage tracing studies have shown that the Pdx1-expressing cells give rise to all three cell lineages in the pancreas, the endocrine, exocrine and duct cells.

Recent advances in analysis and identification of early endodermal or pancreatic genes is remarkable [5-9]. Several reports have demonstrated the identification of novel endodermal genes using early embryos. Embryonic stem (ES) cells are also a highly useful tool in the study of endodermal development. ES cells are pluripotent cells and can be cultured indefinitely in an undifferentiated state, and stimulated to differentiate into various cell types. Progress in ES cell studies has demonstrated that ES cells provide a good system for studies of developmental biology, in addition to the use of ES cells as a surrogate cell source for regenerative medicine. Several groups have reported the differentiation of mouse or human ES cells into the DE or pancreatic lineages [10,11]. We recently established a procedure, where ES cells were cultured on a monolayer of mesodermal-derived M15 cells, and sequentially induced into the mesendoderm, DE and regional specific DE-derived organs *in vitro*. This occurred in a manner that mimics early embryonic inductive events *in vivo *[12,13]. With the addition of activin and bFGF, mouse ES cells differentiated into Pdx1-expressing cells efficiently, reaching 60% of the DE [12]. This M15 procedure turned out not only to be useful in directing DE lineages, but also the ectoderm and mesoderm lineages from ES cells, by altering the culture conditions [14]. Using this M15 differentiation procedure, we tried to validate the differentiated cells by analyzing the expression profiles of three germ layer cells and the pancreatic progenitor cells [14-16]. Comparison of the expression profiles between the cells of the three germ layers, which are derived from the ES cells based on this M15 procedure, revealed a clear distinct clustering of the genes specifically expressed in each germ layer. Studies of Pdx1-exprssing cells derived from ES cells led to the discovery of a novel pancreatic progenitor marker, Eppk1 [15,17] and identification of a novel surface marker, DAF1/CD55, expressed in the DE [16]. Therefore, it is feasible to identify genes related to early DE development and pancreatic differentiation by close investigation into the ES cell-derived cells.

Here we describe an extensive gene expression profile analysis of ES cell-derived definitive endodermal cells and Pdx1-expressing cells. We chose candidate genes by the comparisons between ES cell-derived *Pdx1*-positive or negative DE with the undifferentiated ES cells, ES cell-derived ectoderm, mesendoderm and mesoderm cells. Then we carried out whole mount or section *in situ *hybridization using mouse embryos at E8.5 or pancreas at E14.5, respectively. Out of 54 candidate genes examined, 27 candidate genes are expressed in the DE at E8.5 and 15 genes are expressed in the pancreatic bud at E14.5. These results indicate that the ES cell-derived differentiated cells serve as good models for studies of candidate genes during early embryogenesis.

## Results

### Microarray analysis of ES cell-derived DE cell lineages

**Figure 1:**
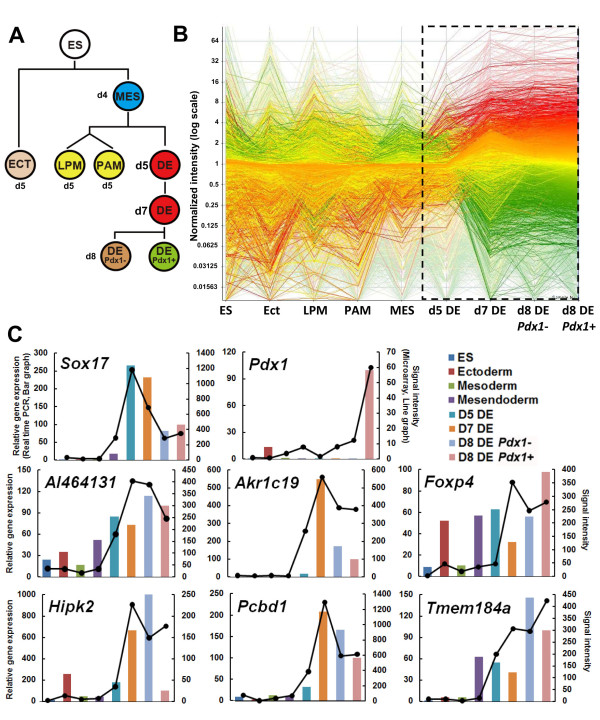
**Microarray analysis of ES cell-derived cells**. (A) ES cells and ES cell-derived cells were isolated by the expression of cell surface antigen as previously described [14]. Populations isolated were: ES cells (ES), ectoderm (ECT), mesendoderm (MES), lateral plate mesoderm, (LPM); Paraxial mesoderm, (PAM) and DE at day 5 (D5), day 7 (D7) and day 8 (D8, DE Pdx1-, DE Pdx1+). (B) Clustering of gene expression in ES cells, ECT, LPM, PAM, MES, d5DE, d7DE, d8DE Pdx1- and d8DE Pdx1+ cell lineages. Each line indicates an individual gene. Red lines indicate genes with high expressions and green lines indicate genes with low expressions in the DE lineages. Y axis represents normalized value of the expression level. (C) Microarray results and real time PCR analyses of six representative candidate genes, namely, *AI464131, Akr1c19, Foxp4, Hipk2, Pcbd1*, and *Tmem184a*, together with *Sox17*, and *Pdx1*, as positive controls. Right Y-axis values represent GeneChip signal intensities of candidate genes. Transcription levels of the representative candidate genes in each population are quantified by real-time PCR analysis. The transcription levels are normalized with that of b-actin. The values are further normalized with that of Pdx1-positive DE on d8 (d8DE GFP+) and thus the left Y-axis values represent relative gene expression levels when the expression level at d8 (d8DE GFP+) is defined as 100.

As previously described, we developed an efficient procedure using mesodermal-derived M15 cells as feeder cells with the supplementation of activin and bFGF, to sequentially differentiate ES cells into, mesendoderm (MES) at day 4 (d4), DE (E-cadherin+/CXCR4+ populations) (d5 to d7) and then Pdx1-expressing cells (d8), (Figure 1A) [12]. ES cells were also differentiated into the three germ layers under different conditions (Shiraki et al. 2009). Using these procedures, ES cell-derived differentiated cells of the ectoderm (SSEA1-/Flk1-/PGFRα-) (ECT), MES (E-cadherin+/PDGFRα+), lateral plate mesoderm (E-cadherin-/PDGFRα-/Flk1+) (LPM), paraxial mesoderm (E-cadherin-/PDGFRα+/Flk1-) (PAM) and DE at d5, d7 and d8 were prospectively isolated, by the expression of specific cell surface antigens using flow cytometry. DE cells at d8 were further subdivided into *Pdx1*/GFP-negative and -positive populations. The efficiencies of the induction of individual populations were similar to that previously reported (Additional file 1). The values were as follows: MES (12%; Additional file 1A), LPM (6%; Additional file 1B), PAM (42%; Additional file 1B), d5DE (45%; Additional file 1C), d5ECT (52%; Additional file 1D), d7DE (53%, Additional file 1E), d8DE (67%), and d8DE Pdx1(-) (35%x67% = 23%; Additional file 1F), or d8DE Pdx1+ (65%x67% = 42%; Additional file 1F). Since ECT was done by negative selection, we confirmed the purity of the ECT using a *Sox1*/GFP ES cell line [14,18]. Ninety-four percent of the sorted ECT turned out to be *Sox1*/GFP-positive neuroectoderm. RNA was extracted from these cells and analyzed by Affymetrix DNA microarray. Then, gene expression profiles of undifferentiated ES cells (ES), ES cell-derived ECT, LPM, PAM, MES, d5DE, d7DE, Pdx1- d8DE and Pdx1+ d8DE, were compared. A remarkable transition of the gene expression profile was observed from d5DE to d7DE and thereafter (Figure 1B).

To validate the microarray results, we selected six representative genes, which showed increased expression levels at >7 fold changes in d8DE, compared to the median signal intensities of other populations, namely ES, ECT, LPM, PAM, MES and d5DE. We examined the actual expression patterns of these genes in ES cell-derived cells. Figure 1C shows the signal intensities of 6 genes: *AI464131*, *Akr1c19*, *Foxp4*, *Hipk2*, *Pcbd1 *and *Tmem184a*, together with those of *Sox17*, an endodermal gene, and *Pdx1*, in each indicated population of ES cell-derived cells obtained in the microarray (Figure 1C). Then, transcription levels of the above genes were quantified by real time PCR analysis (For primer sequences, see Additional file 2). The patterns of the quantitative PCR results correlated well with the signal intensities obtained in the microarray analyses (Figure 1C). The high expression level of *Sox 17 *in d5DE, d7DE and *Pdx1 *in d8DE *Pdx1*+ population, further confirmed the characters of the ES cell-derived cell populations.

### Identification of DE specific genes in ES cell-differentiation

To identify genes involved in DE and pancreatic development, we chose genes that showed raw expression levels above 50 and increased expression in d5DE or d8DE Pdx1+ more than 5 fold change compared to the median signal intensity of ES, ECT, LPM, PAM, MES, d5DE and d8DE Pdx1+. As a result, 165 probe sets (127 genes) or 780 probe sets (594 genes) showed increased transcripts at d5DE and d8DE Pdx1+, respectively, with 115 probe sets (84 genes) overlapping between the two populations. Thus, 50 probe sets (43 genes) were specifically up regulated at d5DE, and decreased thereafter. One hundred and fifteen probe sets (84 genes) were up regulated through d5DE to d8DE Pdx1+. Six hundred and sixty-five probe sets (594 genes) were up regulated at d8DE Pdx1+.

**Figure 2:**
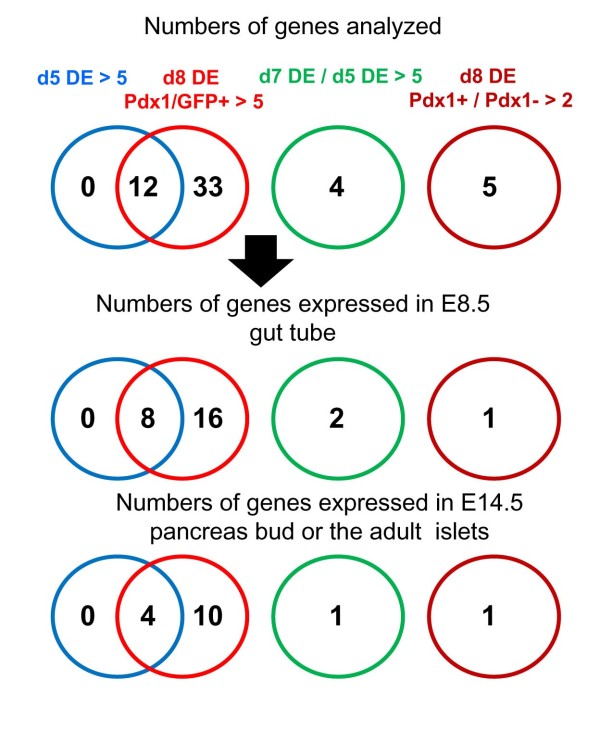
**Numbers of endoderm specific candidate genes**. Summary of the numbers of genes picked up for further analyses by whole mount *in situ *hybridization (top). Numbers of genes that are expressed in gut endoderm at E8.5 (middle) or the pancreatic bud at E14.5 (bottom). Blue circle: genes expressed at >5 fold in d5DE; Red circle: genes expressed at >5 fold in d8DE Pdx1+; Green circle: genes expressed at >5 fold in d7DE versus d5DE; Brown circle, genes expressed at >2 fold in d8DE Pdx1+ versus d8DE Pdx1-

We then examined the expression pattern of some of these DE specific genes in early stages of development. Unknown genes, or genes with specific domain structures, transcription factors or genes whose expression in the endoderm or early pancreas development have not been described, were chosen and analyzed by whole mount *in situ *hybridization. Figure 2 shows the summary of the numbers of genes analyzed and the genes, which were positive in the expression in the gut endoderm at embryonic day (E) E8.5 (or E9.5 for *Apoe*) and/or the pancreatic bud at E14.5 (Figure 2). Of the 115 probe sets (84 genes) that were up regulated at >5 fold at d5 & d8DE Pdx1+, 12 genes were picked, 8 genes were expressed in the E8.5 gut epithelium, and 4 genes were expressed in E14.5 pancreas bud (Figure 2). Of the 665 probe sets (510 genes) that were up regulated at >5 fold at d8DE, 33 genes were examined, 16 genes were expressed in the E8.5 gut epithelium, and 10 genes were expressed in the E14.5 pancreatic bud. Additionally, we randomly picked 4 genes whose expression increased at d7DE at > 5 fold compared to d5DE, and 5 genes that were expressed at d8DE in the Pdx1+ population at >2 fold compared to the Pdx1- population. Of the genes examined, 2 and 1 genes were expressed in the E8.5 gut epithelium, respectively, and 2 (1+1) genes were expressed in E14.5 pancreas bud (Figure 2).

### Genes up regulated in ES cell-derived DE cells are also expressed in the gut endoderm at E8.5

**Figure 3:**
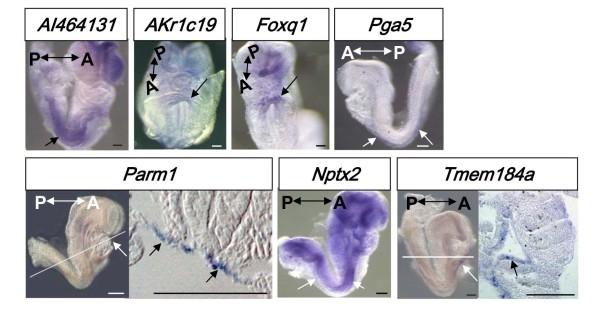
**The expression pattern of 7 candidate genes, whose expression is increased at d5DE in E8.5 mouse definitive endoderm, analyzed by *in situ *hybridization**. *In situ *hybridization pictures of *AI464131*, *Akr1c19*, *Foxq1*, *Pga5*, *Parm1*, *Nptx2 *and *Tmem184a *are shown. Arrows indicate the signals detected. Cross sections through the white line of *Parm1 *and *Tmem184a *are shown (right panels). A: anterior; P: posterior. Scale bars: 100 μm

As shown in Figure 2, out of 12 genes examined, the expression of 8 genes, which increased at >5 fold at d5DE through d8DE, were expressed in E8.5 or E9.5 endoderm. The 8 positive genes were: *AI464131*, *Akr1c19*, *DAF1/CD55*, *Foxq1*, *Nptx2*, *Pga5*, *Parm1 *and *Tmem184a*. Their expression patterns at E8.5 are shown in Figure 3. *AI464131 *and *Nptx2 *were expressed in the whole gut endoderm throughout the anterior-to-posterior region (Figure 3). *Akr1c19 *was expressed weakly in the anterior intestinal portal (AIP, depicted by an arrow) at E8.5, and in the liver bud, pancreas and intestine epithelium at E9.5 (SH data not shown). *DAF1/CD55 *expression is observed in the lateral gut and AIP at E8.5, which was published earlier [16] (Table 1). Similarly, *Tmem184a *was expressed in the lateral gut and AIP at E8.5. *Foxq1 *was expressed strongly in the AIP. *Parm1 *was expressed in the dorsal anterior gut epithelium. *Pga5 *was expressed weakly in the whole lateral gut epithelium. The regions positively detected by *in situ *hybridization are marked by arrows (Figure 3). Cross sections were made and representative results of *Parm1 *and *Tmem184a *expressions are shown (Figure 3).

**Table 1:** A summary of the genes described in this work.

	Gene	Description	Genbank	expression	expression domain at E14.5		Publication on gut or pancreas	
							
				E8.5, endo			Expression or function	Publication
d5 DE > 5 and d8 DE > 5, compared with ES, ECT, LPM, PAM, Mesendoderm, d5 DE	**AI464131**	expressed sequence AI464131	BG063189	whole gut	**epithelium**	**mesenchyme**	―	―
	
	**Akr1c19**	aldo-keto reductase family 1, member C19	BG073853	AIP	**epithelium**		―	―
	
	DAF1/CD55	decay accelerating factor 1	NM_010016	AIP, lateral gut			endoderm	Cell Struct. Func. 2010
	
	Foxq1	forkhead box Q1	NM_008239	AIP			stomach	Gastroenterogy, 2008
	
	Nptx2	neuronal pentraxin 2	NM_016789	whole gut			pancreatic cancer	Cancer Epidemiol Biomarkers Prev, 2008
	
	**Parm1**	Riken cDNA 9130213B05 gene	NM_145562	anterior endoderm	**tip**		E10.5 pancreas	BMC Dev. Biol., 2007
	
	Pga5	pepsinogen 5, group I	NM_021453	lateral gut			―	―
	
	**Tmem184a**	transmembrane protein 184a	BC019731	AIP, lateral gut	**epithelium**		E12.5~, pancreas exocrine	Dev Dyn., 2009

d8 DE, Pdx1(GFP)+> 5, compared with ES, ECT, LPM, PAM, Mesendoderm, d5 DE	**APOE**	apolipoprotein E	AK019319	visceral endoderm		**vascular**	―	―
	
	**C2cd4b**	C2 calcium-dependent domain containing 4B	AK014341	AIP, posterior gut	**trunk**		associated with β-cell function	Diabetologia.,2010
	
	Chi3l1	chitinase 3-like 1	BC005611	AIP			―	―
	
	CpM	carboxypeptidase M	AK004327	AIP, lateral gut			lung	Am. J. Respir. Cell Mol. Biol. 1993
	
	**Creb3l1**	cAMP responsive element binding protein 3-like 1	BC016447	-	**epithelium**	**mesenchyme**	―	―
	
	Fam188b	RIKEN cDNA C330043M08 gene	BB667136	AIP			―	―
	
	Fhl2	four and a half LIM domains 2	NM_010212	AIP, anterior gut			―	―
	
	**Foxp4**	forkhead box P4	BQ286886	AIP, lateral gut	**epithelium**		E9.5~, pulmonary, gut	Mech Dev. 2002
	
	**Hipk2**	homeodomain interacting protein kinase 2	NM_010433	AIP	**epithelium**		E12.5~, pancreas	Endocrinology, 2009
	
	Irf6	interferon regulatory factor 6	NM_016851	anterior gut, hindgut			―	―
	
	Lbh	Limb-bud-and-heart	NM_029999	AIP			―	―
	
	Palld	2410003B16Rik, Ig domain Palladin	NM_001081390	dorsal gut			―	―
	
	**Pbxip1**	pre-B-cell leukemia transcription factor interacting protein 1	AV220340	AIP	**trunk**		―	―
	
	**Pcbd1**	pterin 4 alpha carbinolamine dehydratase/dimerization cofactor of hepatocyte nuclear factor 1 (TCF1)	NM_025273	AIP	**epithelium**			―
	
	**Pcdh1**	protocadherin 1	AK008111	-	**tip**	**mesenchyme**	E12.5~ blood vessels of the gut	Dev Dyn.,2008
	
	**Sox4**	SRY-box containing gene 4	AI428101	AIP, lateral gut	**epithelium**		E12.4~, pancreas	Dev Dyn.,2003
	
	**Tcf7l2**	transcription factor 7-like 2, T-cell specific, HMG-box	BM218908	AIP, lateral gut	**epithelium**	**mesenchyme**	diabetes risk gene	Nat. Genet.2006
	
	Zmiz1	zinc finger, MIZ-type containing 1	NM_183208	AIP, lateral gut				―

d7 DE/d5 DE > 5	**Aebp2**	AE binding protein 2	BB667191	whole gut	**epithelium**		―	―
	
	Barhl2	BarH-like 2 (Drosophila)	NM_001005477	lateral gut			―	―

d8 DE Pdx1(GFP)+/Pdx1(GFP)- > 2	Kiss1r	KISS1 receptor	NM_053244	lateral gut			mouse islets	Diabetologia.,2006

All genes up regulated >5 folds in d5DE, d7DE or d8DE, and their expressions are observed in E8.5 endoderm or E14.5 pancreatic buds are listed. Descriptions, Genbank number, expression patterns in E8.5 endoderm, E14.5 pancreatic buds, and previous publications are summarized.

**Figure 4:**
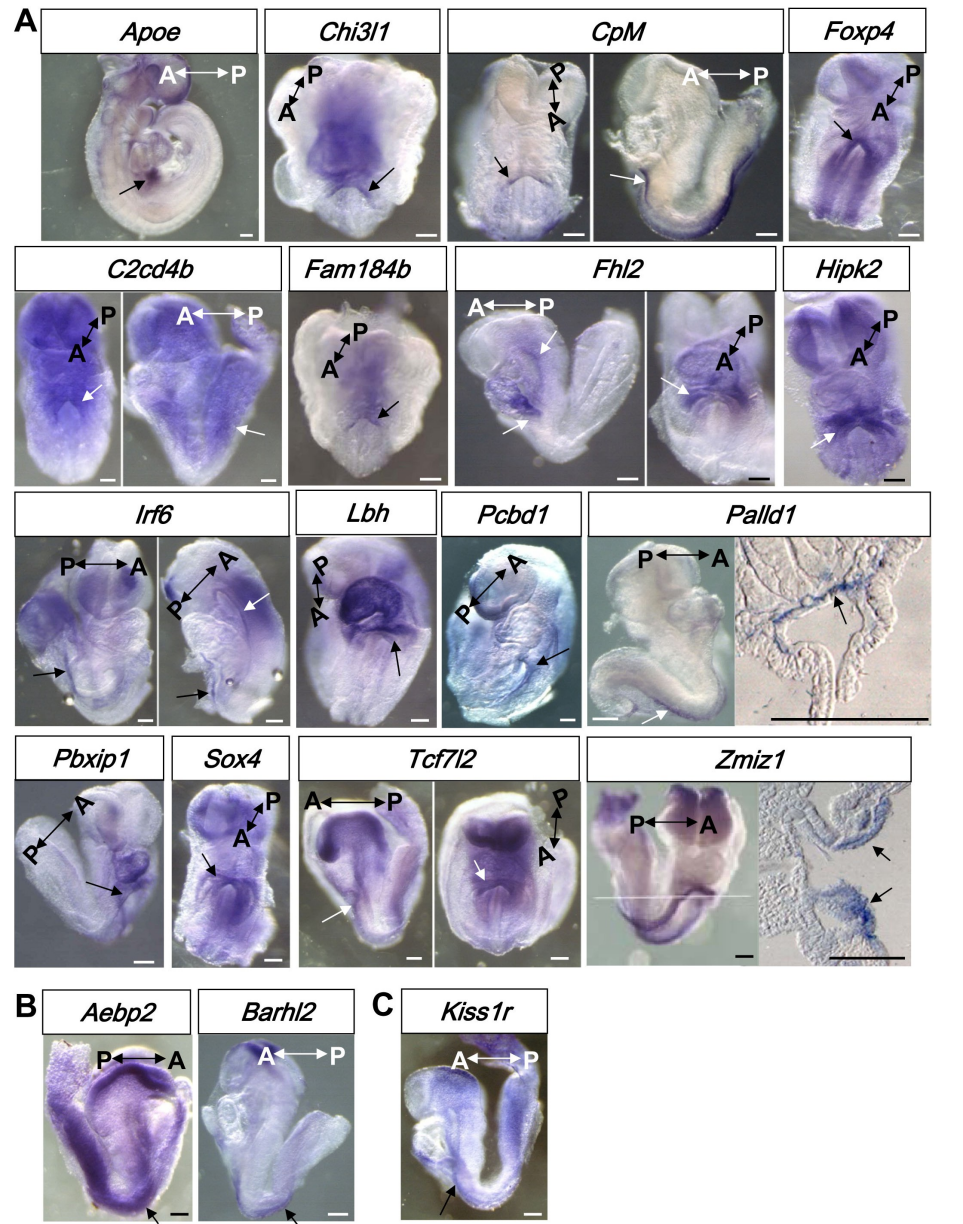
**The expression pattern of 18 candidate genes, whose expression is increased, at d8DE, in E8.5 mouse definitive endoderm, analyzed by *in situ *hybridization**. (A) The expression of 16 candidate genes, whose expression is increased >5 fold in d8DE Pdx1/GFP+ population, (B) >5 fold in d7DE versus d5DE, or (C) >2 fold in d8DE Pdx1+ versus Pdx1- cells, are shown. Gene names are indicated at top of the pictures. Arrows indicate the sites detected. Cross sections through the white line of *Palld1 *or *Zmiz1 *are shown (right panels). A: anterior; P: posterior. Scale bars: 100 μm

The expression patterns by *in situ *hybridization in E8.5 (or E9.5 with *Apoe *gene) of 16 positive genes, whose expression increased first at d8DE are shown in Figure 4A. *Apoe *was expressed in the lateral endoderm and visceral endoderm at E8.5 (SH unpublished) and strongly in the liver bud at E9.5 (Figure 4A, *Apoe*, arrow). Most genes, including *Chi3l1*, *CpM*, *C2cd4b*, *Fam184b*, *Fhl2*, *Foxp4*, *Hipk2*, *Lbh*, *Pcbd1*, *Pbxip1*, *Sox4, Tcf7l2 *and *Zmiz1*, were expressed in the anterior intestinal portal (AIP) in E8.5 embryos (Figure 4A, arrows). In addition to the AIP, *C2cd4b *was also expressed in the hindgut; *CpM*, *Foxp4 *and *Zmiz *were also expressed in the lateral gut epithelium. *Irf6 *was expressed in the anterior endoderm and hindgut epithelium. The lateral gut epithelium expression of *Zmiz1 *was shown by sectioning the E8.5 embryo after *in situ *hybridization (Figure 4A, arrows). An Ig domain gene, *Palld1*, was expressed in the dorsal gut epithelium, also confirmed by sectioning the E8.5 embryo after *in situ *hybridization (Figure 4A, arrow).

The followings are the expression patterns of the genes that were up regulated at d7DE or d8DE, at >5 fold compared to d5DE (Figure 4B) or at a >2 fold increase in Pdx1+ versus Pdx1- population (Figure 4C): Among the 9 genes examined (Figure 2B, Table 1), *Aebp2*, *Barhl *and *Kiss1r *were expressed in E8.5 embryos. *Aebp2 *was strongly expressed in the whole gut tube, and *Barhl2*, *Kiss1r *in the lateral gut epithelium (Figure 4B,C, arrows).

All genes described in Figure 3 and 4 are summarized in Table 1, with their gene descriptions, Genbank number, expression patterns in E8.5 endoderm, and previous publications on expression or function in the endoderm or pancreas. Although they have been implicated in the function of pancreas or other endodermal derived organs, this study showed, for the first time, the expression of many genes in the early stage of development.

### Genes expressed in distinct pancreas domains at E14.5

**Figure 5:**
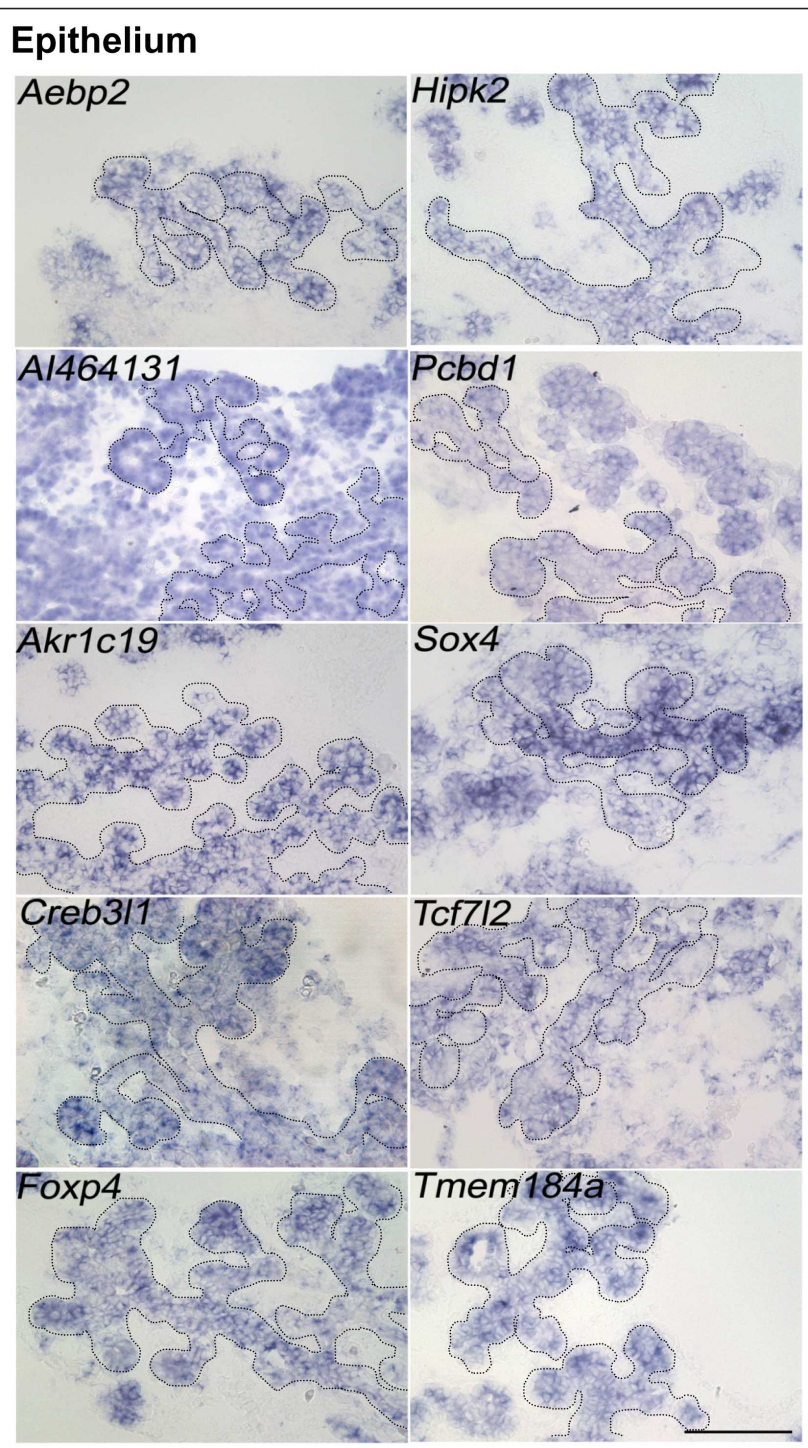
**The expression of 10 candidate genes in E14.5 pancreas epithelium**. *Aebp2*, *AI464131*, *Akr1c19*, *Creb3l1*, *Foxp4*, *Hipk2*, *Pcbd1*, *Sox4*, *Tcf7l2 *and *Tmem184a *are expressed in the pancreas epithelium, detected by *in situ *hybridization. Parts of the pancreas epithelia are out lined with dotted lines. Scale bar: 100 μm, applied to all pictures.

**Figure 6:**
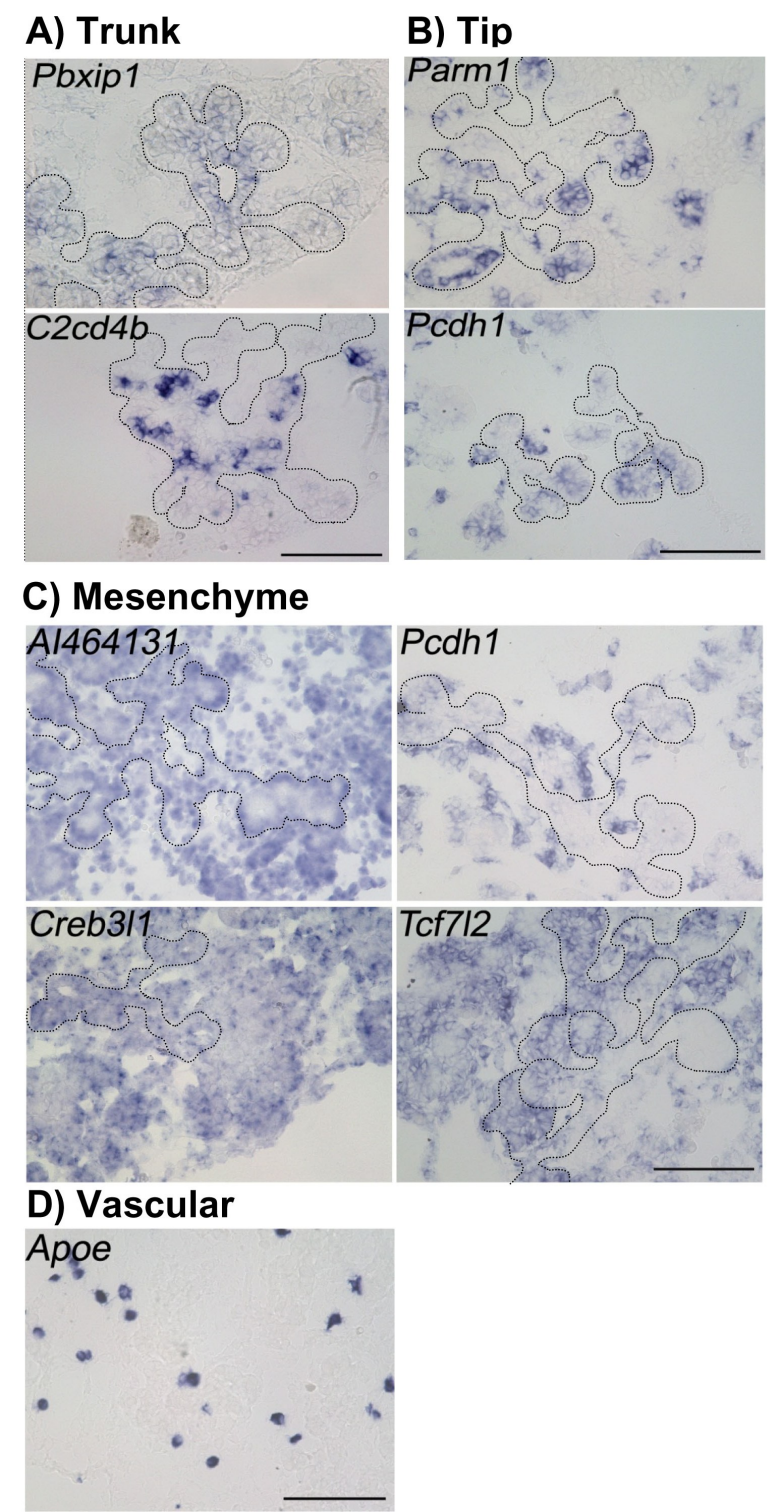
**The expression of candidate genes in E14.5 pancreas distinct regions**. (A) *Pbxip1 *and *C2cd4b *are expressed in the trunk region. (B) *Parm1 *and *Pcdh1 *are in the tip region. (C) *AI464131*, *Creb3l1*, *Pcdh1 *and *Tcf7l2 *are expressed in the mesenchyme. *Pcdh1 *is expressed exclusively in the mesenchyme, whereas the others are also expressed in the epithelium. (D) *Apoe *is expressed in the vascular cells. Parts of the pancreas epithelia are out lined with dotted line in A-C. Scale bars: 100 μm

As described above, of the 54 genes analyzed, 27 genes were expressed in the E8.5 gut epithelium (Figure 3, 4). It was reported that transcripts of genes could be categorized based on their expression patterns into one of five expression domain in the pancreas at E14.5 [5]. We then examined all 54 genes for their expression in E14.5 pancreatic buds. Among the 54 genes, 15 genes are expressed in E14.5 pancreatic buds. The expression patterns were categorized (Figure 5, 6, Table 1). *Aebp2*, *AI464131*, *Akr1c19*, *Creb3l1*, *Foxp4*, *Hipk2*, *Pcbd1*, *Sox4*, *Tcf7l2 *and *Tmem184a *were expressed in the pancreas epithelium (Figure 5). *Pbxip1 *and *C2cd4b *were expressed in the trunk region (Figure 6A). *Parm1 *and *Pcdh1 *were expressed in the tip region (Figure 6B). *AI464131*, *Creb3l1 *and *Tcf7l2 *were also expressed in the mesenchyme, in addition to their epithelial expressions (Figure 6C). *Pcdh1 *was expressed only in the mesenchyme (Figure 6C). *Apoe *was expressed in the vascular cells (Figure 6D). The above gene expression domains in E14.5 pancreatic buds are summarized in Table 1.

### Coexpression of the genes with endocrine and exocrine markers in the pancreatic bud

To investigate further into the function of the genes, their co-expression with the endocrine markers, namely insulin and glucagon, or an exocrine marker, amylase, and Pdx1 were examined.

**Figure 7:**
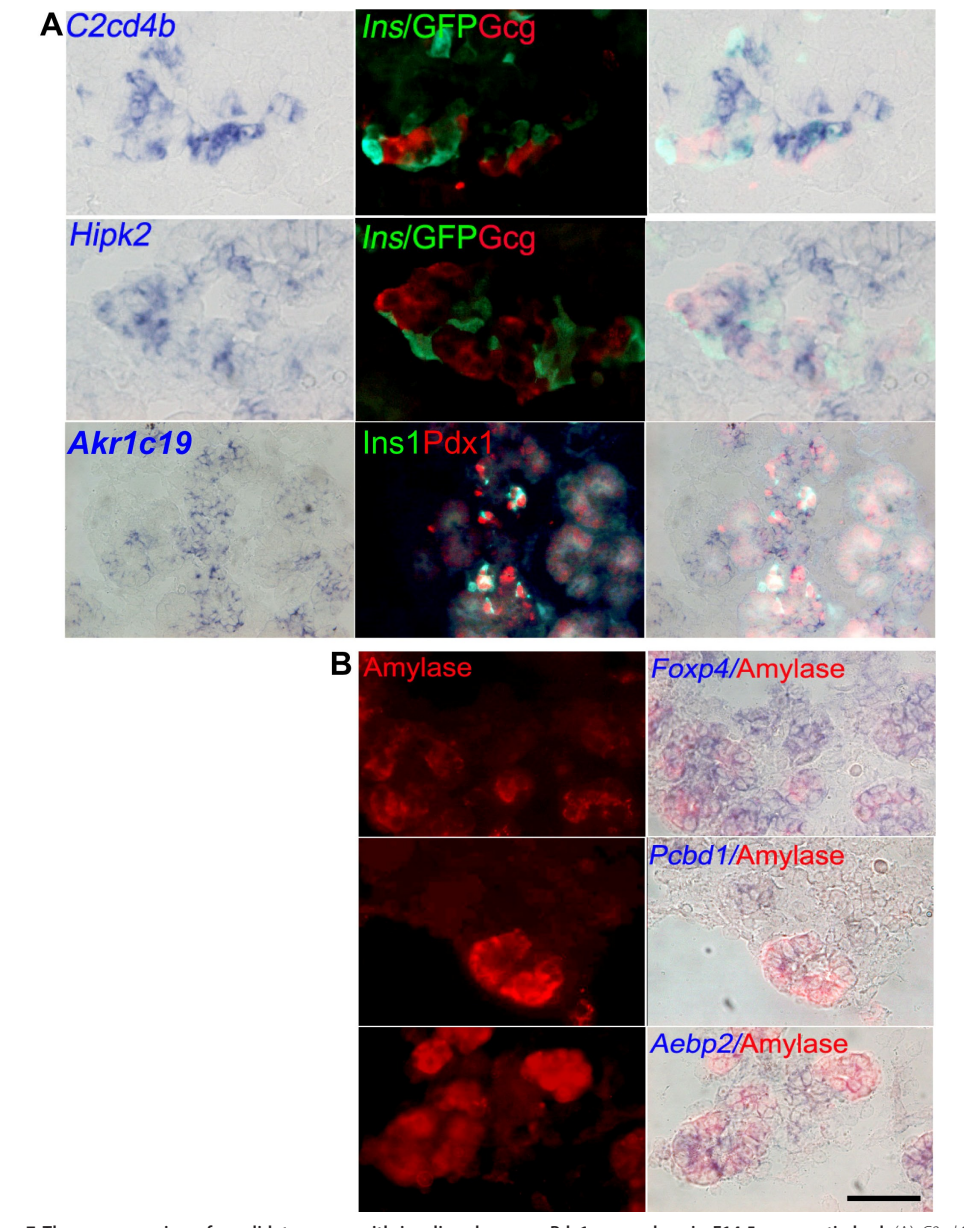
**The co-expression of candidate genes with insulin, glucagon, Pdx1 or amylase in E14.5 pancreatic bud**. (A) *C2cd4b *co-expressed with insulin but not glucagon in the trunk (Upper panels). *Hipk2 *co-expressed with glucagon but not insulin in the epithelium. *Akr1c19 *co-expressed with Pdx1 or insulin in the epithelium. (B) *Foxp4*, *Pcbd1 *and *Aebp2 *partly co-expressed with amylase. Scale bar: 100 μm, applied to A-B.

*C2cd4b*, *a gene *expressed in the trunk (Figure 7A), was co-expressed with insulin, but not glucagon, implicating its function in endocrine β cell differentiation (Figure 7A). *Hipk2 *was co-expressed with glucagon, but not insulin, implicating that it might associate with α cell differentiation (Figure 7A). *Akr1c19 *was co-expressed with Pdx1 or insulin, thus suggesting its possible function in β cell differentiation (Figure 7A). *Foxp4*, *Pcbd1 *and *Aebp2*, which are expressed in the epithelium, were co-localized in part with amylase staining (Figure 7B).

## Discussion

We previously reported the prospective isolation and global expression profiles of the ES cells derived three germ layer cells, such as the mesendoderm, ectoderm, mesoderm and DE, which were obtained by culturing ES cells on M15 [14]. Therefore, M15 provides a platform to compare expression profiles of different lineages derived from ES cells.

In our previous analyses, without the addition of activin and bFGF, but with M15 feeder cells, ES cells derived into immature DE cells, and there were few genes that were expressed in E14.5 pancreatic buds [15]. Here, we used a differentiation protocol with the supplementation of activin and bFGF, a procedure that allows for approximately 50-60% of the differentiated ES cells to give rise to the DE, and among which 60% cells give rise to Pdx1-expressing cells [12,14]. Under the present protocol, ES cells differentiated into a pancreatic developmental pathway. Many genes expressed specifically in the pancreas are identified by comparing d7DE or d8DE with other populations. This may explain the reason for the small differences between GFP+ and GFP- population, since GFP- population are committed to a similar developmental pathway. Using this protocol, we efficiently identified genes specifically expressed in the DE and the pancreatic lineage.

We also examined in our ES cell-derived cells, the expression levels of genes enriched the E7.5 embryo endoderm (Additional file 3) [19], E8.25 endoderm (Additional file 4) [7], and E10.5 Pdx1+ cells (Additional file 5) [19]. Many of the enriched genes in DE of E7.5 embryos were also found to be up-regulated in our ES cell-derived d5DE, d7DE and d8DE GFP+ cells (Additional file 3). Of the endoderm enriched genes in E8.25 embryos, genes such as *Spink3, Clic6, FoxA1, Krt7, Crb3, Ell3, Rab15 Rbm35a, St14 *and *Tmprss2 *are expressed at a higher level in d7DE or d8DE than d5DE. A limited number of E10.5 Pdx1+ cells enriched genes, such as *Mest, Peg3, Col1a2, Tnc, Capn, Gap43 *and *Meis1*, were up regulated in the d8DE GFP+ cells. These results therefore suggest that gene expression profiles in d5, d7 and d8DE were similar with that of E7.5 embryonic DE. However, d7 and d8DE resembled E8.25 embryonic DE compared to d5DE, whereas d8DE resembled E10.5 embryonic Pdx1+ cells compared to the other populations.

### Genes expressed in endodermal tissues other than the pancreas

Among the genes whose expression is increased in the ES cell-derived DE population, several genes are found to be expressed in the foregut, hindgut or whole gut. There are genes previously reported to be expressed in the gut endoderm, or genes with some aspects related to pancreas endocrine or exocrine functions.

*Foxq1*, a fork-head transcription factor, was previously described to be specifically expressed in the stomach in developing and adult gastrointestinal tract. Mice carrying *Foxq1 *mutations show absence of mRNA and protein of the major stomach mucin MUC5AC, and the mutant mice show a paucity of foveolar cell secretory vesicles and a notable loss of stomach mucus [20].

*CpM*, *Carboxypeptidase M*, is a glycosylphosphatidylinositol (GPI)-anchored membrane protein with B-type carboxypeptidase activity and is a member of the 'regulatory' or carboxypeptidase N/E subfamily of metallocarboxypeptidases. *CpM *expression has been described in the developing and adult lung in Alveolar type I cells [21,22]. Recently, the expression of *CpM *was described to be one of the genes expressed in the Foxa2-expressing endoderm cells [23].

*Foxp4*, a member of the forkhead box family of winged-helix genes, is described to be expressed in the pulmonary, neural and gut tissues during embryonic development [24]. It was recently reported to mediate transcriptional repression, by interaction with a component of NuRD, and regulate gene expression and epithelial injury response in the lung via regulation of interleukin-6 [25].

*Pcdh1, Protocadherin-1*, is a member of the δ-protocadherin subgroup of non-clustered protocadherins. The expression is described to be especially prominent in blood vessels in organs derived from the embryonic gut, such as the esophagus, intestines, liver, lung, and submandibular glands [26].

*Zmiz1*, also known as *Zmip10*, is a member of PIAS (protein inhibitor of activated STAT) family of genes. Like the other members of this family, *Zmiz1 *contains the zinc-binding SP-RING Zn-finger domain, which confers SUMO-conjugating activity. At E7.5 *Zmiz1 *transcripts were generally observed in the epiblast, and expression was detected in the neuroectoderm, dorsal aorta and foregut at E8.5 [27]. Taken together, these results suggest that genes up regulated in the ES cell-derived DE at d5 and later, or genes up regulated at d8, are good candidates for being expressed in the gut endoderm at E8.5 during early embryogenesis. Therefore, ES cell-derived DE resembles normal early embryos, and serves as a good source for providing cells for studies on development.

### Genes expressed in the pancreas

Since the differentiation procedure for DE at d7 or d8 is in favor of pancreatic differentiation, there turned out to be many genes expressed in the pancreatic bud. *Parm1*, Riken cDNA 9130213B05, is one of the top ranked 73 genes whose expression is decreased in Pdx1 mutant mice in the E10.5 dorsal pancreatic bud [6]. *Tmem184*, also described as *Sdmg1*, is a component of secretory granules in mouse secretory exocrine tissues, such as pancreas, salivary gland, and mammary gland. Its expression in the developing pancreas was recently reported [28]. homeodomain-interacting protein kinas (HIPK)-2, is expressed in the embryonic and adult pancreas and positively regulates Pdx1 transcriptional activity by directly phosphorylating the C-terminal portion of Pdx1 [29]. *Nptx2 *is one of genes reported with aberrant DNA methylation in familial pancreatic cancers [30].

*Tcf7l2*, also known as *TCF-4*, is a component of the canonical Wnt signaling pathway. A strong genetic association relationship between *Tcf7l2 *polymorphisms and type 2 diabetes mellitus was identified [31]. *Tcf7l2 *was shown to be involved in stimulating the proliferation of pancreatic β-cells and the production of the incretin hormone glucagon-like peptide-1 in intestinal endocrine L cells [32]. Silencing of *TCF7L2 *in human and mouse islets results in a decrease in glucose-stimulated insulin secretion and increased beta cell apoptosis [33,34].

Similarly, variants at *C2cd4b *loci are associated with reduced glucose-stimulated β cell function [35]. *Sox4*, a gene of a member of the homeodomain and basic helix-loop-helix families of transcription factors, is expressed in developing mouse pancreas [36]. *Sox4 *reportedly has a role in insulin secretion in the adult β-cell downstream of the KATP channel, and is identified as a risk factor for diabetes and obesity [37,38]. *Kiss1r*, also known as *GPR54*, is expressed in the islets and in β (MIN5) and α (alphaTC1) cell lines [39]. Kisspeptin, a novel peptide as endogenous ligand of Kiss1r, plays a crucial role in puberty and reproductive function [40], and potentiates glucose-induced insulin secretion from isolated islets [41].

Most genes listed here are revealed for the first time to be expressed at this early stage of E8.5 and E14.5. It is of interest that genes responsible for β-cell maturation are expressed at early stages of development. Future works examining their function would reveal their role in replication or differentiation of the pancreas. These results again highlight the usefulness of the ES cell-derived cells for the discovering of genes that have a role in the development and function of the pancreas.

### Genes found for the first time to be expressed in the definitive endoderm or pancreas

There are genes that have never reported to be expressed in the DE or the pancreas. *Akr1c19 *is an *aldo-keto reductase family 1 member C19 *[42]. *Aebp2 *encodes a zinc finger protein that has been shown to interact with the mammalian Polycomb Repression Complex2 (PRC2) [43]. Its *Drosophila *homolog, *jing*, is a zinc-finger transcription factor that interacts with the fly Polycomb Group (PcG) protein complexes, and plays an essential role in controlling CNS midline and tracheal cell differentiation [44]. *Pbxip1 *is a PBX interacting protein, also known as HPIP, inhibits the binding of Pbx1-Hox complexes to DNA [45]. *Creb3l1*, also known as *OASIS*, is a ZIP (basic leucine zipper) transcription factor, which is a member of the CREB/ATF family, has a transmembrane domain, and has been identified as an ER stress transducer [46]. The physiological functions of these genes in normal pancreatic development remain to be explored.

Finally, there are genes whose expression we could not detect in the Pdx1+ cells during normal pancreatic development. This might be due to their low expression levels and/or technical limitation. In addition, some of the genes show expression patterns that are difficult to be catalogued at E14.5, since the pancreatic differentiation undergoes a secondary transition at this stage and many genes show a dramatic change in their expression patterns after the secondary transition.

## Conclusions

Taken together, our data show that ES cell *in vitro *differentiation is an excellent model system for studies of early developmental processes. ES cell-derived DE cells or Pdx1/GFP-expressing cells obtained from M15 based differentiation procedure mimic cells of the normal developmental processes. Markers identified will be useful for the elucidation of the pancreatic development. This data will also help to derive cells from ES cells that are useful for therapeutics.

## Methods

### Cell lines

The mouse ES cell line, SK7, containing a *Pdx1 *promoter-driven GFP reporter transgene, and the mesonephric M15 cells were cultured and differentiated as previously described [12-14,47].

### Differentiation of ES cells

For mesendoderm (MES), definitive endoderm (DE) differentiation and pancreatic differentiation, ES cells were cultured for 4, 5 or 8 days on M15 added with 20 ng/ml recombinant human activin-A and 50 ng/ml human bFGF, respectively. For neuroectoderm (ECT) differentiation, ES cell culture on M15 was supplemented with 10 μM SB203580 (a MAPK inhibitor). For lateral plate mesoderm (LPM) or paraxial mesoderm (PAM) differentiation, ES cell culture on M15 was supplemented with BMP7 at 25 ng/ml. Isolation of ES cell-derived ECT, MES, and mesoderm (PAM+LPM) cells was performed as previously described [14]. Briefly, MES, DE and mesoderm were isolated as E-cadherin+/PDGFRα+, E-cadherin+/Cxcr4+, and E-cadherin- and Flk1+ or PDGFRα+ fraction, respectively, by cell sorting. Ectoderm cells were isolated as PDGFRα-/Flk1-/SSEA1-. The result of ectoderm differentiation using *Sox1*/GFP ES cells indicated that over 90% of sorted PDGFRα-/Flk1-/SSEA1- cells grown on M15 cell with SB203580 are *Sox1*-positive neuroectoderm cells (Additional file 1).

### Flow cytometry analysis

Cells were dissociated with Cell Dissociation Buffer (Invitrogen, Carlsbad, CA) and adjusted to 1 × 10^6 ^cells/50 μl and stained with appropriate antibodies. The following antibodies were used: biotin- or Alexa 488-conjugated anti-E-cadherin monoclonal antibody (mAb) ECCD-2, biotin- or phycoerythrin (PE)-conjugated anti-CXCR4 mAb 2B11 (BD Pharmingen, San Diego, CA), PE-conjugated anti-SSEA1 mAb (R&D Systems), PE-conjugated anti-FLK1 mAb AVAS12 (BD Biosciences Pharmingen, San Diego, CA), biotin-conjugated anti-PDGFRα mAb APA5 (BD Pharmingen) and streptavidin-allophycocyanin (BD Pharmingen). The stained cells were analyzed using a FACS Canto (BD Pharmingen) or purified with FACS Aria (BD Pharmingen). Data were recorded using the BD FACSDiva Software program (BD Pharmingen) and analyzed using the Flowjo program (Tree Star, Ashland, OR).

### Growth factors and inhibitors

The following concentrations were used: recombinant human activin-A (R&D Systems), 20 ng/ml. Human bFGF (Peprotech), 50 ng/ml; recombinant human BMP7 (R&D Systems), 25 ng/ml; SB203580 (Calbiochem), 10 μM.

### Microarray analysis

The biotinylated cRNAs from differentiated ES cells were hybridized with a MOE430 2.0 series probe array (GeneChip, Affymetrix). The fluorescence intensity of each probe was quantified using the GeneChip Analysis Suite 5.0 computer program (Affymetrix). Data from individual arrays were normalized and expression analysis was performed using the GeneSpring GX software program version 7.3 (Agilent).

### *Quantitative RT *(Reverse transcription) *- real time PCR *(polymerase chain reaction)

RNA was extracted from ES cells using TRI Reagent (Sigma) or RNeasy micro-kit (Qiagen) and then treated with DNase (Sigma). Three micrograms of RNA was reverse-transcribed using MMLV reverse transcriptase (Toyobo) and oligo dT primers (Toyobo). The primer sequences are shown in Additional file 2. The transcript was quantified with SyberGreen on an ABI 7500 thermal cycler (Applied Biosystems). The PCR conditions were as follows: denaturation at 95°C for 15 sec, annealing and extention at 60°C for 60 sec, for up to 40 cycles. Transcripts were normalized to *ß-actin *by subtracting the average *ß-actin *Ct values (Threshold Cycle) from the average Ct of transcripts, resulting in Ct. Target mRNA levels were determined by standard curve method and expressed as arbitrary units.

### In situ hybridization

For whole mount *in situ *hybridization, embryos were fixed with 4% paraformaldehyde overnight, dehydrated in methanol, treated with proteinase K at 10 μg/ml for 10 min, and then refixed in 0.2% glutaraldehyde/4% paraformaldehyde. Hybridization were performed with Digoxigenin (DIG) (Roche) labeled antisense RNA probes at 1 μg/ml. Plasmids for RNA probes were purchased from Open Biosystems or RIKEN BioResource Center (Tsukuba).

For *in situ *hybridization on sections, embryos were fixed with 4% paraformaldehyde overnight, replaced with 30% sucrose in PBS 4˚C overnight and embedded in OCT compound (Sakura Fine technical Co.). *In situ *hybridization with digoxigenin-labeled probes was performed on 10 μm frozen section as described [48,49].

### Immunocytochemistry

The following antibodies were used: goat anti-Amylase (Santa Cruz Biotechnology Inc., Santa cruz, CA), rabbit anti-GFP (MBL International Corp., Woburn, MA), mouse anti-insulin (Sigma-Aldrich), guinea pig anti-glucagon (Linco Research., St.Charles, MO).

## Authors' contributions

SO and SH contributed to acquisition, analysis and interpretation of data. NS contributed to the conception and design, analysis and interpretation of data, and drafting the manuscript. KK carried out interpretation of data, and drafting the manuscript. SK contributed to the conception and design, interpretation of data, financial supports, and drafting the manuscript. All authors critically read, revised and approved the final manuscript.

## Supplementary Material

Additional file 1 **Flow cytometric analyses of ES cell-derived cells of each cell population**. (A) D4 MES (E-cadherin-/PDGFRα+). (B) D5 Mesoderm: PAM (E-cadherin-/PDGFRα+/Flk1-) and LPM (E-cadherin-/PDGFRα+/Flk1-). (C) D5DE (E-cadherin+/CXCR4+), (D) D5 ECT (SSEA1-/Flk1-/PGFRα-), then sorted with Sox1/GFP (X-axis), plotted against FSC (Forward scatter; Y-axis). (E) D7DE (E-cadherin+/CXCR4+), (F) D8DE (E-cadherin+/CXCR4+), then sorted with *Pdx1*/GFP (X-axis), plotted against E-cadherin (Y-axis). Cells collected as the indicated cell population are shown in blue squares (A, left panel in B, C-F), or in the quadrants (right panel in B). The percentages of the collected cell populations are displayed. Click here for file

Additional file 2 **List of primers used for quantitative real-time PCR**. Click here for file

Additional file 3 **A comparisons with E7.5 endoderm enriched genes (versus other germ layers) (Gu et al., Development, 2004)**. Click here for file

Additional file 4 **A comparison with E8.25 endoderm enriched genes (Sherwood et al., Developmental biology, 2007)**. Click here for file

Additional file 5 **A comparison with E10.5 Pdx1+ cells enriched genes (Gu et al., Development, 2004)**. Click here for file
