# Supplementary figures and images for: An expression profile analysis of ES cell-derived definitive endodermal cells and Pdx1-expressing cells
Source: BMC Dev Biol. 2011 Mar 1;11:13. doi: 10.1186/1471-213X-11-13 (PMC3058101; doi:10.1186/1471-213X-11-13)

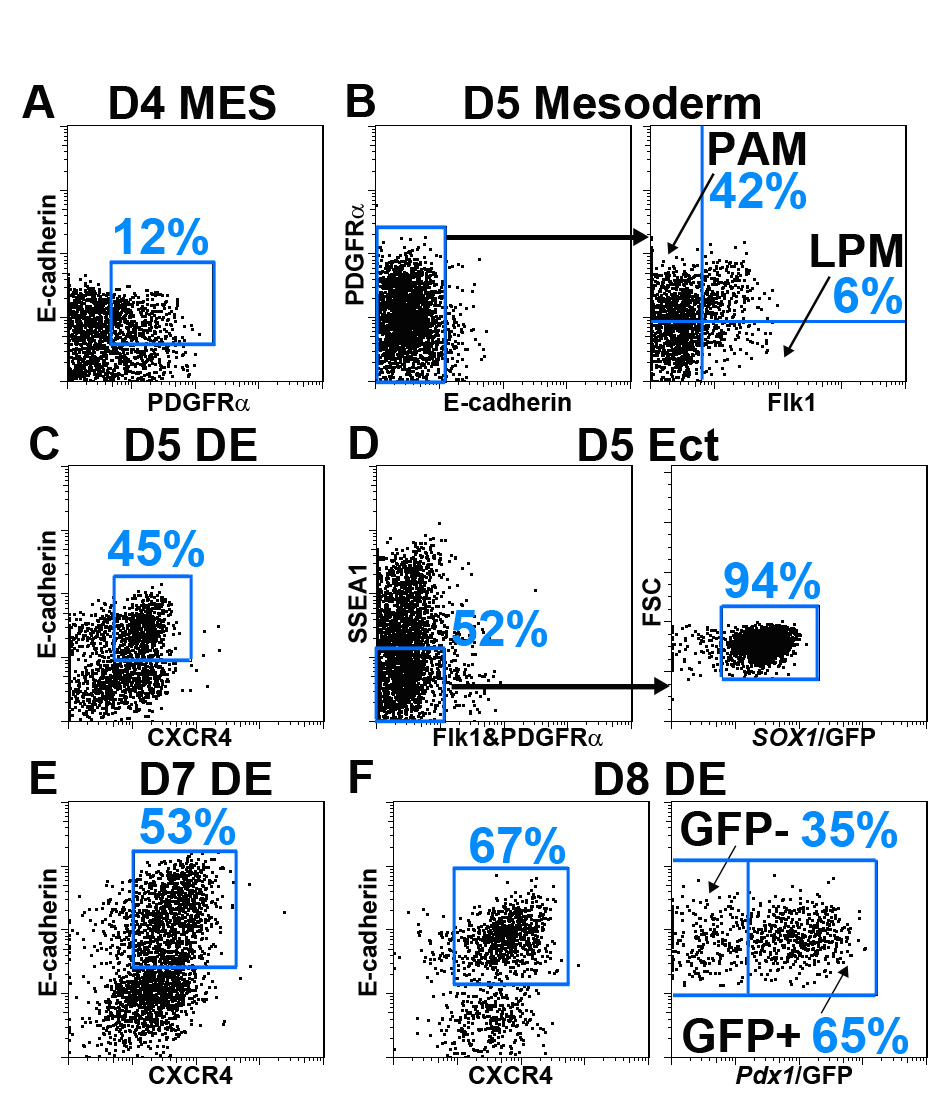

Supplement: Additional file 1 — Flow cytometric analyses of ES cell-derived cells of each cell population. (A) D4 MES (E-cadherin-/PDGFRα+). (B) D5 Mesoderm: PAM (E-cadherin-/PDGFRα+/Flk1-) and LPM (E-cadherin-/PDGFRα+/Flk1-). (C) D5DE (E-cadherin+/CXCR4+), (D) D5 ECT (SSEA1-/Flk1-/PGFRα-), then sorted with Sox1/GFP (X-axis), plotted against FSC (Forward scatter; Y-axis). (E) D7DE (E-cadherin+/CXCR4+), (F) D8DE (E-cadherin+/CXCR4+), then sorted with Pdx1/GFP (X-axis), plotted against E-cadherin (Y-axis). Cells collected as the indicated cell population are shown in blue squares (A, left panel in B, C-F), or in the quadrants (right panel in B). The percentages of the collected cell populations are displayed. [file 1471-213X-11-13-S1.JPEG]
