# Supplementary material for: An expression profile analysis of ES cell-derived definitive endodermal cells and Pdx1-expressing cells
Source: BMC Dev Biol. 2011 Mar 1;11:13. doi: 10.1186/1471-213X-11-13 (PMC3058101; doi:10.1186/1471-213X-11-13)
Supplement: Additional file 2 — List of primers used for quantitative real-time PCR. [file 1471-213X-11-13-S2.PDF]

Additional file 2. PCR primers used to detect gene expressions

Gene	Forward primer	Reverse primer
AI464131	TGTCCTCTGAAATGAGGAACC	TGGAAGGGTTTCTCTGCTGT
Akr1c19	CAGGGGAAGATCTGTTTCCA	ACTTGACCAATCCTGCATCC
β-actin	GTGATGGTGGGAATGGGTCA	TTTGATGTCACGCACGATTTCC
Foxp4	GGAGCTTCTGCACTTCCAAC	GACATCATGGCCACAGACAC
HIPK2	ATCCATGCTGACCTCAAACC	CCACATGTCAATTGCCTCAC
Pcbd1	AGGCCGAGATGCTATCTTCA	ATGGTGGTCCAGCTTTTCAG
Pdx1	CCAAAACCGTCGCATGAAGTG	CTCTCGTGCCCTCAAGAATTTTC
Sox17	GAACAGTTGAGGGGCTACAC	GTTTAGGGTTTCTTAGATGC
Tmem184a	TCACGTTCTTACGCTTCTGC	AGGGTCACGTACAGGTAGCC
