# Supplementary material for: An expression profile analysis of ES cell-derived definitive endodermal cells and Pdx1-expressing cells
Source: BMC Dev Biol. 2011 Mar 1;11:13. doi: 10.1186/1471-213X-11-13 (PMC3058101; doi:10.1186/1471-213X-11-13)
Supplement: Additional file 3 — A comparisons with E7.5 endoderm enriched genes (versus other germ layers) (Gu et al., Development, 2004). [file 1471-213X-11-13-S3.PDF]

Additional file 3 E7.5 endoderm enriched genes (Gu et al., Development, 2004)

A summary of microarray expression in ES, D5 definitive endoderm (DE), D7 DE and D8 DE *Pdx1*/GFP+ for genes enriched in microarrays of E7.5 endoderm versus mesoderm and ectoderm.

-: Flag = absent; +: signal intensity < 300; ++: 300 to 1000; +++: 1000 to 3000; ++++: > 3000.

	ES	D5 DE	D7 DE	D8 DE GFP+
Apoe	+++	++	++++	++++
Car4	+	+++	++++	++++
cer1	-	+++	+++	++++
Cyp26a1	-	+++	++++	++++
Emb	++	++++	++++	++++
Gpc4	+	+++	++++	++++
H19	++	+++	++++	++++
Krt18	+	+++	++++	++++
Krt8	+	++++	++++	++++
Krt8	+	++++	++++	++++
Slc16a1	+++	++++	++++	++++
1600029D21Rik	+	+	++	+++
3110001A13Rik	+	++	+++	+++
ApoA1	-	-	+	+++
Cd59a	+	++	++	+++
Cdkn2b	+	+	+	+++
Cldn6	+	+++	+++	+++
Cnn2	++	+++	+++	+++
Ctsc	+	++	+++	+++
Ctsh	+	++	+++	+++
Ctsz	+++	+++	+++	+++
Dusp1	++	+	++	+++
Fgfbp1	+	+	++	+++
Fhl1	+	++	+++	+++
Flnb	++	++	+++	+++
Fos	+	++	++	+++
Foxa1	-	++	+++	+++
Gab1	+++	++	+++	+++
Itm2b	++	+++	+++	+++
Krt19	+	+	+++	+++
Phlda1	+	+	++	+++
Prss12	-	++	+++	+++
Rbp4	+	+	++	+++
Rdx	+++	+++	++	+++
Sat1	++	++	++	+++
Serpinh1	++	++	+++	+++
Slc39a8	+	+++	+++	+++
Srgn	+	+	++	+++
Trh	+	++++	+++	+++
Vcl	++	++	+++	+++
9130213B05Rik	-	+	+++	++
AA536743	+	+	++	++
Apbb2	+	+	++	++
APP	+	++	++	++
Baiap2l1	+	+	+	++
Cited1	-	+	+++	++
Clcn3	+	++	++	++
Cldn4	+	-	+	++

	ES	D5 DE	D7 DE	D8 DE GFP+
Cldn7	+	++	+++	++
Col4a2	+	+	++	++
Csrp1	+	++	++	++
Dab2	+	+	+	++
Dkk1	+	++	+++	++
Foxa2	-	++	++	++
Foxa3	+	+	++	++
Ggta1	+	++	++	++
Gsn	+	+	++	++
Kdelr3	+	+	++	++
Lcp1	+	+	+	++
Lgmn	+	++	++	++
Lhx1	-	++	++	++
Lrp10	+	+	++	++
Pcbd1	+	+	+++	++
Pdia5	+	++	++	++
Perp	+	++	++	++
Polg	+	++	++	++
Pvrl2	+	+	++	++
Rab5c	+	+	++	++
Rad51	++	++	++	++
Reep6	+	-	++	++
Reep6	+	-	++	++
Rhpn2	+	+	++	++
Slc7a7	+	++	++	++
Sox17	+	+++	++	++
Stard10	+	++	++	++
Tmprss2	-	+	++	++
Tnc	+	+	+	++
Vil1	+	+	+	++
2610019F03Rik	+	+	+	+
A630007B06Rik	+	+	+	+
AA536749	-	+	+	+
Abcd4	+	+	+	+
Adam19	++	+	+	+
Afp	-	-	-	+
Ahr	+	+	+	+
Alkbh	+	+	+	+
Ambp	-	-	+	+
Amot	+	++	+	+
Apoa4	-	-	+	+
Apob	+	-	+	+
APOC2	+	-	+	+
Atf6	+	+	+	+
Capn6	-	-	-	+
Casp4	-	-	+	+
Cdcp1	+	+	+	+
Cdkn1c	+	+	+	+

Chrd	-	+	+	+
COL18A1	+	+	+	+
Col4a1	-	+	++	+
Cpd	+	+	+	+
Cpn1	+	+	+	+
Cubn	+	-	-	+
Cxcr7	+	++	+	+
Dmd	+	+	+	+
Dsc2	-	+	+	+
Dsc2	-	+	+	+
Dscr1l2	+	+	+	+
Dsp	+	+	+	+
Edem1	+	+	+	+
Edg2	+	+	++	+
Elf3	+	+	+	+
Epha2	+	+	+	+
Esrra	-	-	+	+
Eya2	-	+	+	+
F10	-	-	-	+
Fgg	-	-	+	+
Folr1	++	+	+	+
Foxd4	-	-	-	+
Gadd45b	-	-	+	+
Gas6	+	+	+	+
Gjb3	+	+	+	+
Gpx2	+	-	+	+
Hey1	+	+	+	+
Igf2	-	-	+	+
Igf2r	+	+	+	+
Igfbp5	+	+	+	+
Ilvbl	+	+	+	+
JAK1	+	+	+	+
junB	+	+	+	+
Kitl	-	+	+	+
Klf6	+	+	+	+
Klf6	+	+	+	+
Lamb3	-	+	+	+
Lnx1	+	+	+	+
Myo5b	-	-	-	+
Nedd9	+	+	+	+
Nfkb1	+	+	+	+
Nid2	++	+	+	+
Nr2f2	-	+	+	+
Nr4a1	+	+	+	+
Otud7b	+	+	+	+
Pdzk1	-	-	-	+
Pea15a	+	+	+	+
Plat	+	+	+	+
Pon2	+	+	-	+
Rab11fip5	+	+	+	+
Reck	+	+	++	+

Rhox5	+++	+	+	+
Rrbp1	+	+	+	+
Scel	-	+	+	+
Sipa1	+	+	+	+
Six1	+	+	+	+
Slc2a3	+++	+++	+	+
Slc2a3	+++	+++	+	+
Slc34a2	+	+	+	+
Smoc1	+	+	+	+
Soat2	-	-	+	+
Spef1	-	-	+	+
Tcfcp2l1	++	-	+	+
Tex19	+	+	+	+
Tex264	-	+	+	+
Tfpi	+	+	+	+
Tgtp	-	+	-	+
Tnfrsf21	+	+	+	+
Trap1a	+++	+	+	+
Ttr	-	-	+	+
Ugt1a6a	+	+	+	+
Vtn	-	+	-	+
Wdr26	+	+	+	+
Wnt11	-	+	+	+
Xbp1	+	+	++	+
Zbtb16	-	+	+	+
Abcc2	-	-	-	-
Ahnak	-	-	-	-
Apom	-	-	-	-
Arsa	-	-	-	-
Bhmt2	-	-	+	-
ccl5	-	-	-	-
Cpa3	-	-	-	-
Fgb	-	-	-	-
Hdc	-	-	-	-
Klf5	+	-	-	-
Lgr5	-	-	-	-
Lrig1	-	-	-	-
Ltb	-	-	-	-
Ltb	-	-	-	-
Lyn	+	-	-	-
Mapk12	-	-	-	-
Msx1	-	-	+	-
Nr1h3	-	-	-	-
Pthlh	-	-	-	-
Slc2a2	-	-	-	-
Slc7a9	-	-	-	-
Tacstd2	-	-	-	-
Tekt1	-	-	-	-
Xlr3a; Xlr3b	+	-	+	-
Xlr4b	+	-	-	-
Xpr1	+	+	-	-
