# Supplementary material for: An expression profile analysis of ES cell-derived definitive endodermal cells and Pdx1-expressing cells
Source: BMC Dev Biol. 2011 Mar 1;11:13. doi: 10.1186/1471-213X-11-13 (PMC3058101; doi:10.1186/1471-213X-11-13)
Supplement: Additional file 4 — A comparison with E8.25 endoderm enriched genes (Sherwood et al., Developmental biology, 2007). [file 1471-213X-11-13-S4.PDF]

Additional file 4 E8.25 endoderm enriched genes (Sherwood et al., Developmental biology, 2007)

A summary of microarray expression in ES, D5 definitive endoderm (DE), D7 DE and D8 DE *Pdx1*/GFP+ for 31 genes enriched in microarrays of E8.25 endodermal cells as compared to those on non-endodermal cells (E8.25 extraembryonic mesoderm, embryonic ectoderm, embryonic mesoderm).

-: Flag = absent; +: signal intensity < 300; ++: 300 to 1000; +++: 1000 to 3000; ++++: > 3000.

	ES	D5 DE	D7 DE	D8 DE GFP+
Emb	++	++++	++++	++++
Spink3	+	+++	++++	++++
Clic6	+	++	+++	+++
FoxA1	-	++	+++	+++
Krt7	+	+	++	+++
Tacstd1	++	+++	+++	+++
AnxA4	+	++	++	++
Crb3	+	+	++	++
Ell3	+	+	++	++
Rab15	+	+	++	++
Rbm35a	+	++	+++	++
Sox17	+	+++	++	++
St14	+	+	++	++
Tmem30b	+	+	+	++
Tmprss2	-	+	++	++
Ankrd56	+	+	+	+
Cdcp1	+	+	+	+
Dpp4	+	-	-	+
Dsg2	+	+	+	+
Gprc5c	-	+	+	+
Prss8	+	+	+	+
Ripk4	+	+	+	+
Sh3gl2	+	-	-	+
Bnipl	-	-	-	-
Cacna1b	-	-	-	-
Cldn8	-	-	-	-
Nepn	-	-	-	-
Npnt	-	-	-	-
