# Supplementary material for: An expression profile analysis of ES cell-derived definitive endodermal cells and Pdx1-expressing cells
Source: BMC Dev Biol. 2011 Mar 1;11:13. doi: 10.1186/1471-213X-11-13 (PMC3058101; doi:10.1186/1471-213X-11-13)
Supplement: Additional file 5 — A comparison with E10.5 Pdx1+ cells enriched genes (Gu et al., Development, 2004). [file 1471-213X-11-13-S5.PDF]

Additional file 5 E10.5 Pdx1+ cells enriched genes (Gu et al., Development, 2004)
A summary of microarray expression in ES, D5 definitive endoderm (DE), D7 DE and D8 DE Pdx1/GFP+ for genes enriched in microarrays of E10.5 Pdx1+ cells.
-: Flag = absent; +: signal intensity < 300; ++: 300 to 1000; +++: 1000 to 3000; ++++: > 3000.

	ES	D5 DE	D7 DE	D8 DE GFP+
Mest	++	+++	+++	++++
Peg3	+	+++	+++	++++
Col1a2	+	+	+	++
Erbb3	+	++	++	++
Gas1	+	+	++	++
Itm2a	+	+	++	++
Mki67	++	++	+++	++
Sfrp1	+	+	++	++
Tnc	+	+	+	++
Tnfaip8	+	++	+	++
wsb1	++	++	++	++
6330403K07Rik	+	++	+	+
Aspm	++	+	+	+
AU020206	++	++	+	+
Capn6	-	-	-	+
CDC7	++	++	++	+
Cdkn1c	+	++	+	+
Col1a1	+	+	+	+
Col5a2	+	+	+	+
Dlk1	+	+	++	+
Gap43	+	-	-	+
Gcs1	+	++	++	+
Gja7	+	++	+	+
Igfbp5	+	+	+	+
Meis1	-	-	-	+
Mfap2	-	+	+	+
Mfap2	-	++	+	+
Mgst1	+	++	+	+
Pcm1	+	++	+	+

	ES	D5 DE	D7 DE	D8 DE GFP+
Plagl1	+	+	+	+
Rprm	+	++	+	+
Rprm	+	++	+	+
Snai2	+	+	+	+
Sox11	+	+	+	+
Tanc1	-	-	+	+
Tcf21	-	-	-	+
Unc5c	-	-	-	+
Vnn1	+	+	-	+
1700066C05Rik	+	-	-	-
Acan	+	-	-	-
Barx1	-	-	-	-
Chst2	-	-	-	-
Dnahc11	-	-	-	-
Hba-a1	-	-	-	-
Hba-x	-	-	-	-
Hbb-y	-	-	-	-
Kcnj8	-	-	-	-
Myb	+	++	-	-
Nkx6-2	-	-	-	-
Nr2f1	-	++	-	-
Onecut1	-	-	+	-
Ptn	-	-	-	-
Sez6	-	-	-	-
Sp4	-	-	-	-
Suz12	+	-	-	-
Tac2	-	-	-	-
Thy1	+	-	-	-
Zfp125	-	-	-	-
